# Exosomes Secreted from Hypoxia-Preconditioned Mesenchymal Stem Cells Prevent Steroid-Induced Osteonecrosis of the Femoral Head by Promoting Angiogenesis in Rats

**DOI:** 10.1155/2021/6655225

**Published:** 2021-04-07

**Authors:** Na Yuan, Zhaogang Ge, Wenchen Ji, Jia Li

**Affiliations:** ^1^Department of Ultrasonography, The First Affiliated Hospital of Xi'an Jiaotong University, Xi'an, Shaanxi Province 710061, China; ^2^Department of Sports Medicine, Honghui Hospital of Xi'an Jiaotong University, Xi'an, Shaanxi Province 710054, China; ^3^Department of Orthopedics, The First Affiliated Hospital of Xi'an Jiaotong University, Xi'an, Shaanxi Province 710061, China

## Abstract

Recent studies have suggested that exosomes exert similar therapeutic effects to those of mesenchymal stem cells (MSCs) in regenerative medicine and MSCs-derived exosomes exhibit therapeutic effects on steroid-induced osteonecrosis of the femoral head (ONFH). Furthermore, reparative functions of exosomes from MSCs are enhanced by hypoxia treatment of the cells. However, there are no related reports about whether exosomes derived from hypoxia-preconditioned MSCs could show better therapeutic effects on steroid-induced ONFH. In vitro, we investigated the effects of hypoxia precondition on exosomes derived from bone marrow mesenchymal stem cells (BMMSCs) from rats and the proangiogenic ability of exosomes derived from hypoxia-preconditioned BMMSCs. In vivo, we investigated the role of exosomes from hypoxia-preconditioned BMMSCs on angiogenesis and protecting osteonecrosis in a rat ONFH model. We found that the potential of the proangiogenic ability of exosomes derived from hypoxia-preconditioned BMMSCs was higher than exosomes derived from BMMSCs cultured under normoxia. Exosomes derived from hypoxia-preconditioned BMMSCs significantly promoted proliferation, migration, vascular endothelial growth factor (VEGF) expression, and tube formation of human umbilical vein endothelial cells (HUVECs) compared with exosomes derived from BMMSCs cultured under normoxia. Administration of exosomes derived from hypoxia-preconditioned BMMSCs significantly prevented bone loss and increased vessel volume in the femoral head compared with exosomes derived from BMMSCs cultured under normoxia. Taken together, our data suggest that exosomes derived from hypoxia-preconditioned BMMSCs exert better therapeutic effects on steroid-induced ONFH by promoting angiogenesis and preventing bone loss.

## 1. Introduction

Steroid-induced osteonecrosis of the femoral head (ONFH) is caused by long-term glucocorticoid use. It often affected young patients aged 30-50 years. If the treatment is not timely and properly, approximately 75% of affected hips will progress to femoral head collapse and many of these patients need total hip arthroplasty. Although the underlying molecular mechanisms of the pathogenesis of steroid-induced ONFH remain unclear, it is generally accepted that the final common pathway is the decrease of the vascular supply to the femoral head, resulting in the necrosis of osteocytes and marrow and destruction of the bone architecture.

Bone marrow mesenchymal stem cells (BMMSCs) have multilineage differentiation potential and can promote tissue regeneration and repair. Transplantation of BMMSCs has been regarded as novel methods of ONFH therapy. Recent studies indicated that the paracrine effects of BMMSCs, instead of differentiation, contribute to the positive outcomes of BMMSCs therapy. Paracrine effects are mediated by paracrine factors including cytokines, growth factors, and extracellular vesicles (EVs). Among these paracrine factors, exosomes play a vital role in the beneficial paracrine effects of BMMSCs therapy. Recent studies suggest that exosomes exhibit therapeutic effects similar to those of MSCs [1, 2].

Exosomes are a small subset of extracellular vesicles that range from 40 nm to 150 nm [3]. They are endosome-derived vesicles secreted by most cell types. Exosomes are formed when the endosome membrane invaginates to produce multivesicular bodies (MVBs), which then fuse with the plasma membrane to release exosomes to adjacent cells or into circulation by exocytosis. Exosomes are packed with cell-type-specific coding RNA, noncoding RNA, proteins, and other molecular constituents. Exosomes facilitate cell-cell communication by shuttling these functional cargoes. Thus, exosomes exert special biological characteristics. Recent animal studies have shown that exosomes have therapeutic effects in steroid-induced ONFH. Kuang et al. evaluated the effects of exosomes derived from Wharton's jelly of human umbilical cord mesenchymal stem cells in glucocorticoid-induced ONFH in a rat model, and they found that exosomes are effective in preventing glucocorticoid-induced ONFH [4]. Guo et al. demonstrated that exosomes derived from human synovial-derived mesenchymal stem cells could prevent steroid-induced ONFH in a rat model [5]. Liu et al. studied the effects of exosomes derived from human-induced pluripotent stem cell-derived mesenchymal stem cells in steroid-induced ONFH and they got similar results [6].

Hypoxic precondition of MSCs can promote the proliferation and osteogenic differentiation potential of MSCs and, therefore, improve the therapeutic efficacy of MSCs transplantation for treatment of ONFH [7]. Furthermore, hypoxic precondition attenuates the abnormal osteogenic/adipogenic differentiation of BMMSCs obtained from individuals with ONFH [8]. It has been reported that the contents and functions of exosomes are affected by the cellular environment where the host cells stay. Anderson et al. analyzed the proteinaceous contents of exosomes derived from MSCs cultured under ischemic tissue simulated conditions (1% O_2_) and found that these exosomes showed increased proangiogenic factors which are beneficial for the treatment of ischemic diseases [9]. Han et al. have demonstrated that hypoxia can significantly elevate the beneficial effect of exosomes in the fat grafting by improving the blood perfusion and survival of the grafted tissue [10]. Zhu et al. indicated that hypoxia treatment of MSCs enhanced the myocardial reparative functions of exosomes derived from MSCs in a mouse model of myocardial infarction [11]. Liu et al. found that compared with exosomes derived from MSCs cultured under normoxia, exosomes derived from MSCs cultured under hypoxia promoted angiogenesis, proliferation, and migration of HUVECs to a greater extent [12]. In a mouse model of femoral fracture, they demonstrated that exosomes derived from MSCs cultured under hypoxia exhibited greater therapeutic effects on bone fracture healing.

However, whether exosomes derived from hypoxia-preconditioned BMMSCs could exhibit better therapeutic effects on steroid-induced ONFH was unclear. Based on the above findings, we hypothesized that exosomes derived from BMMSCs cultured under hypoxia might exhibit increased proangiogenic ability and protective effect on steroid-induced ONFH. To validate this hypothesis, in vitro experiments were performed to investigate the effects of hypoxic precondition on exosomes derived from BMMSCs from rats and the proangiogenic ability of exosomes derived from hypoxia-preconditioned BMMSCs. Additionally, an early-stage rat model of steroid-induced ONFH was established by using lipopolysaccharide combined with methylprednisolone which has been proved to be effective in modelling ONFH [13–15], and the therapeutic effect of exosomes derived from hypoxia-preconditioned BMMSCs on steroid-induced ONFH in rats was studied.

## 2. Materials and Methods

### 2.1. Isolation of BMMSCs and Exosomes

Four-week-old Sprague-Dawley rats were used in this study, and all the animals were from the Experimental Animal Center of Xi'an Jiaotong University (Shaanxi, China). Rats BMMSCs were obtained as described previously [14]. Briefly, the femurs and tibias were resected under sterile conditions. Bone marrow was flushed out by repeated aspiration with syringes. After centrifuged at 800 rpm for 5 min, the collected cells were seeded into 6-well dishes and culture in *α*-minimum essential medium (*α*MEM) supplemented with 10% fetal bovine serum (FBS, Gibco, CA, USA) and 1% penicillin-streptomycin (Gibco, CA, USA) in an incubator with 5% CO2 at 37°C. Cells, in passages 3-5, were used in subsequent experiments.

Exosomes were extracted from conditioned media of BMMSCs by differential centrifugations. After cultured in a medium containing 10% exosome-depleted FBS under hypoxia (2% oxygen) or normoxia (20% oxygen) for 48 h, the conditioned media was collected and centrifuged at 300 × g for 10 min, 16500 × g for 20 minutes at 4°C. The supernatant was then filtered through a 0.22 *μ*m filter to remove cellular debris. Exosomes were then pelleted by ultracentrifugation at 120000 × g for 70 min at 4°C. Finally, the pelleted exosomes were resuspended in PBS and stored at -80°C. Exosomes collected from BMMSCs cultured under normoxia and hypoxia were denoted as Exos^N^ and Exos^H^, respectively.

### 2.2. Characterization of Exosomes

The morphology of exosomes was observed by transmission electron microscopy (TEM). The typical surface markers of exosomes, including CD9 and TSG101, and negative protein marker of exosomes including Calnexin were detected by Western blotting. To determine the absolute size distribution and particle concentration of exosomes, nanoparticle tracking analysis (NTA) was performed by using a NanoSight NS300 (Marvel, UK).

### 2.3. Effects of Exos^N^ and Exos^H^ on Human Umbilical Vein Endothelial Cells (HUVECs)

#### 2.3.1. Cell Proliferation Assay

The CCK8 kit (Dojindo, Kumamoto, Japan) was used to determine proliferation according to the manufacturer's protocol. HUVECs were seeded in 96-well plates at the density of 2 × 10^3^ cells/well and treated with Exos^N^ (50 *μ*g/mL), Exos^H^ (50 *μ*g/mL) or PBS. After 2 days, the culture medium was removed, and 100 *μ*L premixed culture medium containing 10 *μ*L of CCK8 was added to each well. The OD values at 450 nm were measured after incubation for another 3 h at 37°C.

#### 2.3.2. Wound Healing Assay

HUVECs at 2 × 10^5^ cells/well were seeded in a 24-well plate and grown to confluence. A straight scratch across each well was made by a sterile micropipette tip. Each well was washed three times with PBS to remove loose cells. Culture medium containing exosomes at 50 *μ*g/mL or PBS in the same volume was added, and the cells were incubated in a 37°C humidified incubator with 5% CO_2_. Wound closure was measured and recorded by using the ImageJ software at 0 h, 12 h, and 24 h.

#### 2.3.3. Transwell Migration Assay

2 × 10^4^ cells resuspended in serum-free medium were seeded into the upper chambers of the transwell chambers (Corning). 600 *μ*L of complete medium containing exosomes at 50 *μ*g/mL or PBS in the same volume was placed in the lower chamber. 12 hours later, the cells on the up surface of the membranes were removed with cotton swap. The migrated cells were fixed with ethanol and stained with crystal violet. The number of migrated cells was counted under a light microscope (Leica).

#### 2.3.4. Enzyme-Linked Immunosorbent Assay (ELISA)

Concentrations of vascular endothelial growth factor (VEGF) in the cellular supernatant of HUVECs were tested by ELISA. Briefly, after the cells were cultured in the medium containing exosomes at 50 *μ*g/mL or PBS in the same volume for 2 days, the cell supernatant was collected and centrifuged to remove cellular debris. The concentrations of VEGF were determined by using the Human VEGF Quantikine ELISA Kit (R&D Systems, Inc., MN, USA) following the manufacturer's instructions.

#### 2.3.5. Tube Formation Assay

To study the effect of Exos^N^ and Exos^H^ on capillary network formation of HUVECs, a tube formation assay was performed. HUVECs were purchased from the American Type Culture Collection (ATCC; Manassas, VA, USA). 2 × 10^4^ cells resuspended in the medium containing exosomes at 50 *μ*g/mL or PBS in the same volume were seeded into matrigel-coated 96-well plate. After incubation for 4 h, tube formation was observed under an optical microscope (Leica), and the number of complete tubes was calculated using the ImageJ software.

#### 2.3.6. Quantitative Real-Time Polymerase Chain Reaction Analysis (qRT-PCR)

After treatment with Exos^N^ (50 *μ*g/mL), Exos^H^ (50 *μ*g/mL), or PBS at the same volume for 2 days, the mRNA levels of VEGFA in the HUVECs were determined by qRT-PCR. The total RNA was extracted with TRIzol reagent according to the instructions (Invitrogen, Carlsbad, CA, USA). RNA was reverse transcribed to cDNA using the PrimeScript RT Master Mix Kit (TaKaRa, Shiga, Japan). The levels of mRNA were measured by the SYBR Premix Ex Taq™ Kit (TaKaRa, Shiga, Japan). GAPDH served as an internal control. The following primers were used: VEGFA, forward: 5′-CGCT CGGTGCTGGAATTTGA-3′, reverse: 5′-AGTGGGGAA TGGCAAGCAAA-3′ and GAPDH, forward: 5′-TATGAC TCTACCCACGGCAAGT-3′, reverse: 5′-ATACTCAGCAC CAGCATCACC-3′. Each sample was analyzed in triplicate.

### 2.4. Effects of Exos^N^ and Exos^H^ on Rats Steroid-Induced ONFH

#### 2.4.1. ONFH Model and Treatments

The Animal Ethical Committee of the Xi'an Jiaotong University approved all procedures that were carried out. Sprague-Dawley rats weighing 240–280 g were used. Forty-eight healthy male rats were randomly divided into four groups (*n* = 12 per group): (1) control group, the rats were treated with normal saline; (2) model group, the rats with steroid-induced ONFH; (3) Exos^N^ group, the rats with steroid-induced ONFH were treated with Exos^N^; (4) Exos^H^ group, the rats with steroid-induced ONFH were treated with Exos^H^. Steroid-induced ONFH model was established in the model group, Exos^N^ group, and Exos^H^ group. Rats ONFH model was established according to published protocol [13]. 4 mg/kg body weight lipopolysaccharide (LPS, Sigma, St. Louis, MO, USA) were intravenously injected twice at a time interval of 24 h. After 24 hours, the rats were injected three times with 60 mg/kg body weight of methylprednisolone (MPS, Pfizer, New York, USA) at a time interval of 24 h. A sham injection with normal saline was performed in the control group. After the last MPS injection, the rats in Exos^N^ group and Exos^H^ group were intravenously injected with 100 *μ*g exosomes (Exos^N^ and Exos^H^, respectively) every week for 4 weeks. The rats in model group were given an equal volume of PBS. Four weeks after the last injection of MPS, animals were used for further study.

#### 2.4.2. Histopathology and Immunohistochemistry

Four weeks after the last injection of MPS, rats in each group were sacrificed and bilateral thighbones were obtained for HE staining and immunohistochemistry.

For HE staining, the femoral heads were fixed with 10% neutral buffered formalin for 24 hours at 4°C. Then, the samples were decalcified with 10% ethylenediaminetetraacetic acid (EDTA) for 6 weeks, dehydrated with ethanol, embedded in paraffin, cut along the coronal plane into 4 *μ*m-thick sections, and stained with hematoxylin-eosin. The sections were observed under a light microscope.

To observe angiogenesis in the femoral head, immunohistochemical analysis for expression levels of VEGF was performed. Briefly, after the samples were cut along the coronal plane into 4 *μ*m-thick sections, the sections were deparaffinized in xylene and rehydrated in ethanol. Then, the sections were immersed in 3% H_2_O_2_ for 10 min to block endogenous peroxidase activity and blocked with 10% fetal bovine serum for 30 min. The sections were, respectively, further incubated with anti-VEGF primary antibody overnight at 4°C and incubated with biotinylated secondary antibody for 30 min. Subsequently, the sections were treated with diaminobenzidine solution to develop the coloring and stained with hematoxylin. The negative control sections were treated with PBS instead of the primary antibody. The sections were observed under a light microscope and the images were captured using the same settings.

#### 2.4.3. Micro-CT Scanning

Four weeks after the last injection of MPS, the femoral heads were obtained and fixed with 10% neutral buffered formalin for 24 hours. Then, bone microstructure of the femoral heads was analyzed by micro-CT (eXplore Locus SP, GE, USA). The resolution of the scanner was 14 *μ*m per pixel. Image reconstruction was performed using a Reconstruction Utility. Region of interest (ROI) was selected in the femoral head. Quantitative parameters of the trabecular bone including bone mineral density (BMD), bone volume/total volume of bone (BV/TV), trabecular number (Tb.N), and trabecular separation (Tb.Sp) were analyzed.

#### 2.4.4. Vessel Analysis

To observe vessels in the femoral head, micro-CT-based vessel analysis was performed four weeks after the last injection of MPS. After the rats were anesthetized, a silicone rubber injection compound (Microfil MV-122; Flow Tech, Carver, MA, USA) was perfused as previously reported [13]. After the rats were stored at 4°C overnight, the bilateral femoral heads were obtained and decalcified in 10% EDTA for 6 weeks. Then, the samples were subjected to micro-CT scanning. The blood vessel volume was analyzed.

### 2.5. Statistical Analysis

All experiments were repeated at least three times. All the data were displayed as means ± SEM. The Student's *t*-test was applied to compare two groups, and one-way analysis of variance was applied for multigroup comparisons. Data analysis was performed with SPSS 18.0. All the data demonstrated a normal distribution between groups checked by the Shapiro-Wilk test. A *p* value less than 0.05 was considered significant.

## 3. Results

### 3.1. Characterization of Exosomes

**Figure 1:**
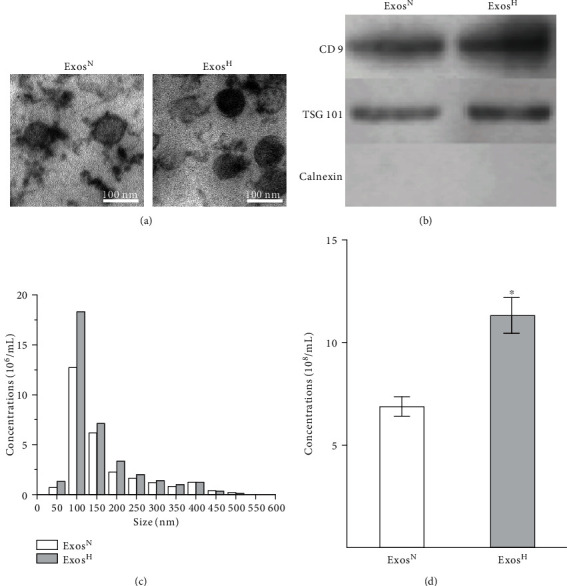
Characterization of Exos^N^ and Exos^H^. (a) Typical cup-shaped morphology assessed by TEM. (b) Western blot analysis of CD9, TSG101, and Calnexin expression in Exos^N^ and Exos^H^. (c) The size distribution of Exos^N^ and Exos^H^ assessed by nanoparticle tracking analysis. (d) The concentration analysis of Exos^N^ and Exos^H^ assessed by nanoparticle tracking analysis. The results are from three independent experiments. The data are expressed as the means ± SEMs. ^∗^*p* < 0.05 compared with the Exos^N^ group.

TEM, western blotting for the typical surface markers of exosomes, and nanoparticle tracking analysis were used to characterize Exos^N^ and Exos^H^. TEM images showed that both Exos^N^ and Exos^H^ exhibited classic cup-shaped morphology ranging from 40 to 150 nm in diameter (Figure 1(a)). Western blot analysis indicated that both Exos^N^ and Exos^H^ were positive for CD9 and TSG101, which are typical surface markers of exosomes (Figure 1(b)). Neither Exos^N^ nor Exos^H^ expressed Calnexin, which is a negative protein marker of exosomes. Nanoparticle tracking analysis (Figure 1(c)) showed that both Exos^N^ and Exos^H^ exhibited similar size distribution (approximately 100 nm). The results of TEM and nanoparticle tracking analysis indicated no morphological difference between Exos^N^ and Exos^H^ was observed. However, the exosome concentration (Figure 1(d)) assessed by nanoparticle tracking analysis was increased in Exos^H^ compared with Exos^N^ (*p* < 0.05), indicating that hypoxia treatment induced release of exosomes.

### 3.2. Exos^H^ Promote Proliferation and Migration of HUVECs In Vitro

**Figure 2:**
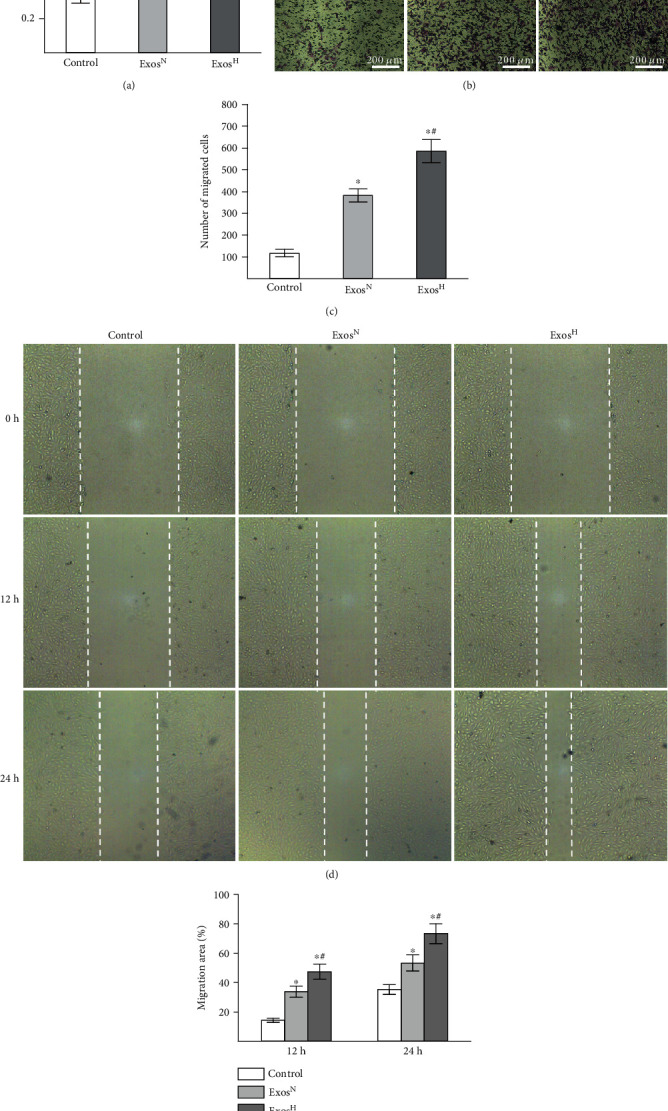
The effect of Exos^N^ and Exos^H^ on proliferation and migration of HUVECs. (a) Proliferation of HUVECs treated with Exos^N^ and Exos^H^ detected by CCK-8 assay. (b) Representative images of transwell assay showing the migrated HUVECs. (c) Quantitative analysis of transwell assay. (d) Representative images of wound healing assay. (e) Quantitative analysis of wound healing assay. The results are from three independent experiments. The data are expressed as the means ± SEMs. ^∗^*p* < 0.05 compared with the control group; ^#^*p* < 0.05 compared with the Exos^N^ group.

To explore the effect of Exos^N^ and Exos^H^ on the proliferation of HUVECs, CCK8 proliferation assay was performed. As shown in Figure 2(a), both Exos^N^ and Exos^H^ enhanced the proliferation of HUVECs compared with the control group (*p* < 0.05). However, Exos^H^ treatment group showed increased proliferation than that of the Exos^N^ group (*p* < 0.05).

Transwell migration assay and wound healing assay were used to examine the effect of Exos^N^ and Exos^H^ on the migration of HUVECs. As shown in Figures 2(b) and 2(c), results of transwell assay indicated that both Exos^N^ and Exos^H^ promoted the migration of HUVECs compared with the control group (*p* < 0.05). However, the migratory capacity of HUVECs in Exos^H^ group was significantly greater than those in the Exos^N^ group (*p* < 0.05). Similar results were observed in wound healing assay (Figures 2(d) and 2(e)). Taken together, these results indicated that Exos^H^ remarkably promoted the proliferation and migration of HUVECs.

### 3.3. Exos^H^ Promote Angiogenic Activity of HUVECs In Vitro

**Figure 3:**
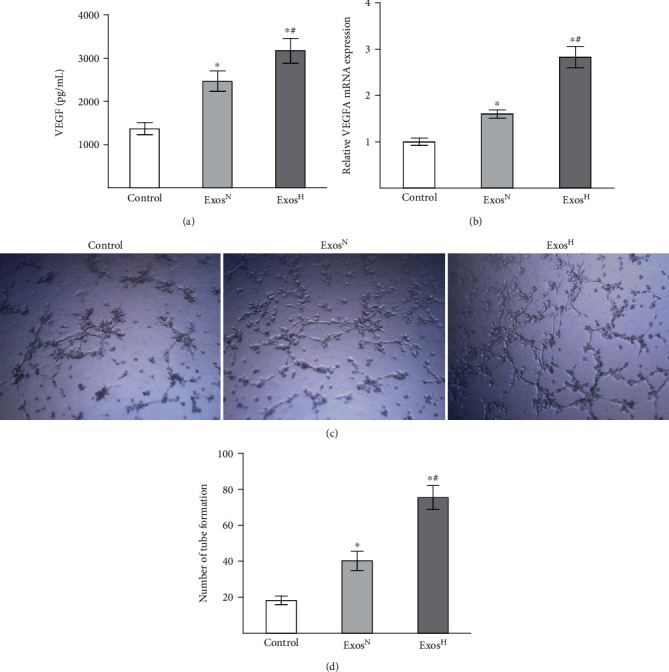
Exos^H^ promotes angiogenic activity of HUVECs. (a) Level of VEGF in the supernatant was measured by ELISA. (b) Level of VEGFA mRNA was measured by qRT-PCR. (c) Capillary-like structures formation by HUVECs cultured with Exos^N^ and Exos^H^. (d) Quantitative analysis of tube formation assay. The results are from three independent experiments. The data are expressed as the means ± SEMs. ^∗^*p* < 0.05 compared with the control group; ^#^*p* < 0.05 compared with the Exos^N^ group.

As VEGF is critical for angiogenesis, the concentrations of VEGF in the supernatant were measured by ELISA, and the expression of VEGFA was measured by qRT-PCR after HUVECs were treated with Exos^N^ and Exos^H^. The ELISA results (Figure 3(a)) showed that both Exos^N^ and Exos^H^ promoted VEGF secretion compared with the control group (*p* < 0.05). However, HUVECs treated with Exos^H^ showed higher VEGF secretion than those of the Exos^N^ group (*p* < 0.05). As shown in Figure 3(b), both Exos^N^ and Exos^H^ contributed the mRNA level of VEGFA (*p* < 0.05), and the Exos^H^ group displayed a higher mRNA level of VEGFA than the Exos^N^ group (*p* < 0.05).

The effect of Exos^N^ and Exos^H^ on tube formation of HUVECs was evaluated in an in vitro matrigel assay. The results of the tube formation assay indicated that compared with the control group, both Exos^N^ and Exos^H^ promoted capillary-like structure formation in HUVECs (Figure 3(c)). However, quantitative analysis of tube formation assay (Figure 3(d)) showed that the number of tube formation was significantly elevated in Exos^H^ group than those in the Exos^N^ group and the control group (*p* < 0.05). Collectively, these results indicated that the angiogenic activity of Exos^H^ in vitro was greater than Exos^N^.

### 3.4. Exos^H^ Protect Osteonecrosis of the Femoral Head in a Rat ONFH Model

**Figure 4:**
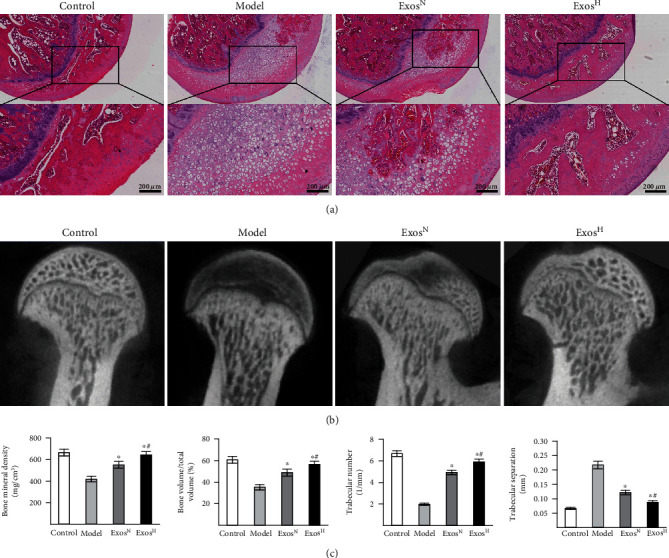
Exos^H^ protect bone tissue in a rat ONFH model. (a) HE staining of rat femoral heads. (b) Micro-CT scanning images. (c) Quantitative analysis of micro-CT. Each group consisted of 5 femoral heads (*n* = 5). The data are expressed as the means ± SEMs. ^∗^*p* < 0.05 compared with the model group; ^#^*p* < 0.05 compared with the Exos^N^ group.

To study the effects of Exos^H^ on osteonecrosis of the femoral head in vivo, a rat ONFH model was established. Bone tissues of the femoral head were observed by HE staining (Figure 4(a)) and micro-CT scanning (Figure 4(b)). Histological results based on HE staining indicated that no osteonecrosis was observed in the normal group. In the model group, ONFH changes were apparent. The femoral head in the model group showed sparser trabecular bone and the trabecular bone was even replaced by necrotic tissues. However, in Exos^N^ group, slight osteonecrosis of the trabecular bone was observed. The trabecular bone in Exos^N^ group was well arranged, and few trabecular bone and bone marrow were replaced by necrotic tissues. Furthermore, in the Exos^H^ group, no osteonecrosis was observed and the trabecular bone maintained a better structure and structural integrity.

The micro-CT results were consistent with the histological findings. Quantitative analysis of micro-CT (Figure 4(c)) indicated that the microstructural parameters of the trabecular bone including BMD, BV/TV, Tb.N, and Tb.Th in the model group were worse compared with those in the control group (*p* < 0.05). After additional treatment with Exos^N^, these parameters were reversed in the Exos^N^ group compared with those in the model group (*p* < 0.05). In addition, these parameters were further reversed in the Exos^H^ group compared with those in the Exos^N^ group (*p* < 0.05).

**Figure 5:**
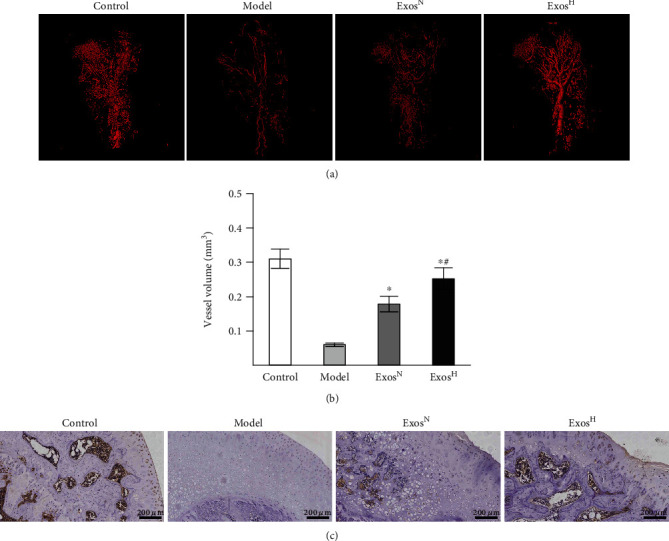
Exos^H^ promotes angiogenesis in the femoral head in a rat ONFH model. (a) Micro-CT-based microangiography analysis. (b) Quantitative analysis of vessel volume. (c) Immunohistochemical staining for VEGF. Each group consisted of 5 femoral heads (*n* = 5). The data are expressed as the means ± SEMs. ^∗^*p* < 0.05 compared with the model group; ^#^*p* < 0.05 compared with the Exos^N^ group.

Micro-CT-based microangiography and immunohistochemical staining for VEGF were performed to evaluate angiogenesis of the femoral head. As shown in Figure 5(a), the vessel structure in the model group was impaired compared with those in the control group. After additional treatment with Exos^N^ or Exos^H^, such impairment was reversed. Quantitative analysis of vessel volume indicated that the vessel volume was significantly increased in the Exos^H^ group compared with the model group and the Exos^N^ group (Figure 5(b)). Expression of VEGF, which is an important angiogenic factor and usually indicates potential angiogenic function, was analyzed by immunohistochemical staining. Immunohistochemical staining results showed that the VEGF expression decreased in the model group compared with that in the control group, and VEGF expression increased in the Exos^H^ group compared with the model group and the Exos^N^ group (Figure 5(c)).

These results indicated that Exos^H^ prevents osteonecrosis of the femoral head by protecting bone tissue and promoting angiogenesis.

## 4. Discussion

In the present study, we investigated the effects of hypoxia preconditioning on the biological properties of BMMSCs-derived exosomes in vitro. Our results revealed that exosomes collected from BMMSCs cultured under hypoxia significantly promoted proliferation, migration, VEGF expression, and tube formation of HUVECs compared with exosomes collected from BMMSCs cultured under normoxia. Moreover, we explored for the first time the role of exosomes from hypoxia-preconditioned BMMSCs on angiogenesis and protecting osteonecrosis in a rat ONFH model and our results showed that exosomes collected from BMMSCs cultured under hypoxia exerted a better proangiogenic and bone-protective effects than exosomes collected from BMMSCs cultured under normoxia.

Although MSCs transplantation has great potential in promoting bone repair and regeneration, there are some disadvantages. One disadvantage of MSCs transplantation is the low survival rate of transplanted stem cells in ischemic tissues [16, 17]. Moreover, other important disadvantages of MSCs transplantation are undesired immune response, tumorigenicity, and cell dedifferentiation [18]. In addition, increasing evidence indicated that the transplanted MSCs exert their therapeutic effects mainly by their paracrine activity, and it is now well known that the paracrine activity of MSCs is due to their ability to release extracellular vesicles including exosomes [19].

Recent studies indicated that exosomes exert similar therapeutic effects to those of MSCs in regenerative medicine [20, 21]. Furthermore, exosome-based therapy avoids all the problems caused by direct MSCs transplantation and displays several advantages over direct MSCs transplantation. Exosomes have low immunogenicity and have no risk of tumor formation because of their inability to self-replicate. They have no vascular obstructive effect and have good permeability because of their small size, which make them easily move into wound areas and capillary [22–24]. These features make exosome-based therapy a promising treatment option for tissue repair.

Many studies have shown that MSC-derived exosomes exert protective effects in bone regeneration and repair by stimulating angiogenesis. In an ovariectomized rat model of critical-sized calvarial defects, Qi et al. found that exosomes secreted by MSCs derived from human-induced pluripotent stem cells promote bone repair by enhancing angiogenesis and osteogenesis [25]. Takeuchi et al. demonstrated that exosomes derived from conditioned media of BMMSCs promote bone regeneration in a rat model of calvaria bone defect by enhancing angiogenesis [26]. Qin et al. evaluated the effects of bone marrow stromal cell-derived extracellular vesicles in the regulation of osteoblast activity and bone regeneration. In their study, the authors demonstrated that bone marrow stromal cell-derived extracellular vesicles positively regulate osteoblastic differentiation in vitro and stimulate bone formation in the critical-size calvarial bone defects of rat [27]. In a rat model of stabilized fracture, Zhang et al. showed that exosomes derived from human umbilical cord mesenchymal stem cells promote fracture healing by promoting angiogenesis [28]. As described above, several studies have demonstrated the beneficial effects of exosomes in osteonecrosis of the femoral head. In a rat model of steroid-induced ONFH, Liu et al. demonstrated that exosomes secreted by induced pluripotent stem cell-derived mesenchymal stem cells show a preventative effect on osteonecrosis of the femoral head by promoting local angiogenesis and preventing bone loss [6]. In another study, Guo et al. demonstrated that exosomes secreted by human synovial-derived mesenchymal stem cells also have therapeutic effects on glucocorticoid-induced ONFH [5]. Kuang et al. showed that exosomes derived from Wharton's jelly of human umbilical cord mesenchymal stem cells can prevent rat glucocorticoid-induced ONFH by inhibiting osteocyte apoptosis [4]. Tao et al. discovered that exosomes derived from human platelet-rich plasma could prevent rat ONFH by inhibiting apoptosis induced by glucocorticoid-associated endoplasmic reticulum stress [29]. In our study, we demonstrated that MSC-derived exosomes could protect osteonecrosis in a rat ONFH model. Results of micro-CT-based vessel analysis showed that MSC-derived exosomes could promote angiogenesis of the femoral head.

Exosomal functions and components are highly affected by cell microenvironment where the host cells stay. Several approaches have been taken to improve exosomal functions and modify exosomal components including hypoxia preconditioning, serum deprivation, and genetic modifications of exosome-producing cells. Li et al. have transfected BMMSCs with HIF-1a, leading to the secretion of exosomes that showed an increased expression of HIF-1a [30]. They found that exosomes secreted from HIF-1a-modified BMMSCs showed increased proosteogenic and proangiogenic effects in vitro. In addition, they injected the HIF-1a-enriched exosomes into the necrosis region of the femoral in a rabbit steroid-induced avascular necrosis of femoral head model and found that the bone regeneration and angiogenesis were accelerated in vivo. Zuo et al. transfected human CD34+ stem cells with miR-26a and got similar results in vitro and in a rat glucocorticoid-induced ONFH model [31].

It was revealed that exosomes originating from hypoxic MSCs have an increased levels of proangiogenic factors compared to exosomes originating from normoxic cells [9]. By protein array analysis, Han et al. found that exosomes released by adipose mesenchymal stem cells under hypoxic condition showed increased levels of vascular endothelial growth factor (VEGF), epidermal growth factor (EGF), fibroblast growth factor (FGF), and their receptors (VEGF-R2, VEGF-R3) compared to exosomes released by normoxic cells [32]. Furthermore, they demonstrated exosomes originating from hypoxic human adipose-derived mesenchymal stem cells enhance angiogenesis through VEGF/VEGF-R in the nude mice model of fat grafting. Xue et al. found that exosomes released by hypoxia-exposed human adipose mesenchymal stem cells can improve angiogenesis by activating the protein kinase A signaling pathway [33]. Conducted by Liu et al., miRNA microarray results of exosomes derived from hypoxic MSCs showed an upregulation of miR-126 which has been proven to play important roles in angiogenesis. In addition, they found that exosomes derived from hypoxic MSCs promote bone fracture healing by the transfer of miR-126 in a mouse femoral fracture model [12]. Up to now, the therapeutic effects of exosomes derived from hypoxia-preconditioned BMMSCs in steroid-induced ONFH have not been studied. In our study, we found that exosomes derived from hypoxia-preconditioned BMMSCs significantly promoted proliferation, migration, VEGF expression, and tube formation of HUVECs compared with exosomes derived from normoxic BMMSCs. Furthermore, we found that exosomes derived from hypoxia-preconditioned BMMSCs showed better therapeutic effects on steroid-induced ONFH with promoted angiogenesis and bone regeneration.

In the present study, there are still some limitations. Although we compared the biological properties between exosomes from hypoxia-preconditioned BMMSCs and exosomes from BMMSCs cultured under normoxia, the detailed molecular differences of the components between the two different exosomes have not been investigated. In addition, the exact molecular mechanism of the promoted therapeutic effects of exosomes derived from hypoxia-preconditioned BMMSCs in steroid-induced ONFH has not been investigated. Furthermore, the number of replicates was small (*n* = 3 or *n* = 5) in our study which may limit the significance of this study. Thus, further studies are needed to address these limitations.

In conclusion, our studies suggested that exosomes derived from hypoxia-preconditioned BMMSCs possess superior effects to exosomes derived from BMMSCs cultured under normoxia in promoting angiogenesis in vitro. In addition, exosomes derived from hypoxia-preconditioned BMMSCs exert increased beneficial effects on steroid-induced ONFH by enhancing angiogenesis in vivo. This study may be helpful for providing a new strategy to prevent the development of steroid-induced ONFH.

## Data Availability

The datasets supporting the conclusions of this article are included within the article and available from the corresponding author on reasonable request.

## Abbreviations

ATCC: American Type Culture Collection
BMD: Bone mineral density
BMMSCs: Bone marrow mesenchymal stem cells
BV/TV: Bone volume/total volume of bone
EDTA: Ethylenediaminetetraacetic acid
ELISA: Enzyme-linked immunosorbent assay
EVs: Extracellular vesicles
FBS: Fetal bovine serum
HUVECs: Human umbilical vein endothelial cells
LPS: Lipopolysaccharide
MPS: Methylprednisolone
MSCs: Mesenchymal stem cells
MVBs: Multivesicular bodies
NTA: Nanoparticle tracking analysis
qRT-PCR: Quantitative real-time polymerase chain reaction analysis
ROI: Region of interest
ONFH: Osteonecrosis of the femoral head
TbN: Trabecular number
TbSp: Trabecular separation
TEM: Transmission electron microscopy
VEGF: Vascular endothelial growth factor
*α*MEM: *α*-Minimum essential medium.
